# Assessment of markers of primary aldosteronism in systemic sclerosis and their relationships with renal and cardiovascular outcomes

**DOI:** 10.1136/rmdopen-2026-006930

**Published:** 2026-07-15

**Authors:** Aurore Collet, Sébastien Sanges, Stéphanie Espiard, Bodale Djobo, Romain Vankemmel, Sylvain Dubucquoi, Eric Hachulla, Claire Douillard, David Launay, Jun Yang, Fabien B Vincent

**Affiliations:** 1 INSERM, CHU Lille, Institute for Translational Research in Inflammation (INFINITE), University of Lille, Lille, France; 2 INSERM, Lille, France; 3 Institute of Immunology, CHU Lille, Lille, France; 4 Département de Médecine Interne et Immunologie Clinique, CHU Lille, Lille, France; 5 NordOuest, Méditerranée et Guadeloupe (CeRAINOM), Centre National de Référence Maladies Auto-immunes Systémiques Rares du Nord, Lille, France; 6 Health Care Provider of the European Reference Network on Rare Connective Tissue and Musculoskeletal Diseases (ReCONNET), Lille, France; 7 Department of Endocrinology and Metabolism, CHU Lille, Lille, France; 8 Service Hormonologie, Métabolisme, Nutrition, Oncologie, Pôle de Biologie Pathologie Génétique, CHU Lille, Lille, France; 9 Centre for Endocrinology and Reproductive Health, Hudson Institute of Medical Research, Clayton, Victoria, Australia; 10 Department of Medicine, Monash University, Clayton, Victoria, Australia; 11 Rheumatology Research Group, Centre for Inflammatory Diseases, Department of Medicine, School of Clinical Sciences at Monash Health, Monash University, Clayton, Victoria, Australia

**Keywords:** Autoimmune Diseases, Biomarkers, Cardiovascular Diseases, Hypertension, Scleroderma, Systemic

## Abstract

**Background:**

Whether primary aldosteronism (PA), the most common endocrine cause of hypertension (HTN), contributes to HTN and cardiovascular disease burden in systemic sclerosis (SSc) has never been explored. We aimed to assess the prevalence and management of HTN, prevalence of positive PA screening test results and the associations between markers of PA with HTN and SSc-related outcomes in SSc.

**Methods:**

Data from adult SSc patients and normotensive healthy controls (HC) were analysed in this single-centre study. HTN was defined as blood pressures ≥140/90 mm Hg on two visits or as documented in medical records. PA screening was performed using the aldosterone:renin ratio (ARR). Associations between biomarkers of PA (renin, aldosterone, ARR, hybrid steroid 18-hydroxycortisol (18OHF)) with HTN and SSc-related outcomes were assessed using multivariable regression modelling.

**Results:**

Data from 112 SSc patients (median (IQR) age 60 (49, 69) years, 79% female) and 48 HC (median (IQR) age 42.5 (31, 57) years, 52% female) were analysed. At baseline, 41 SSc patients (37%) had HTN, increasing to 52% of the cohort at the last follow-up visit (median follow-up 5.1 years). The prevalence of positive baseline ARR in SSc was 8% (9/112), corresponding to 12% (5/41) in the hypertensive subset. No associations were observed between biomarkers of PA and SSc-related renal and cardiovascular outcomes or HTN at last follow-up visit.

**Conclusions:**

HTN was common in this SSc cohort, and prevalence of positive PA screening test was estimated at 12% among hypertensive SSc patients, supporting consideration of PA screening in this population.

**WHAT IS ALREADY KNOWN ON THIS TOPIC**

- Despite the prevalence of hypertension in systemic sclerosis (SSc), its underlying pathophysiology remains incompletely understood. Limited evidence suggests a link between primary aldosteronism (PA) and autoimmune diseases; however, the relationship between PA, hypertension and disease outcomes in SSc has not previously been examined.

**WHAT THIS STUDY ADDS**

- This study provides the first evaluation of the prevalence of positive PA screening results in SSc, which is comparable to that seen in the general hypertensive population. However, biomarkers of PA were not independently associated with hypertension, renal involvement or cardiovascular outcomes in this cohort.

**HOW THIS STUDY MIGHT AFFECT RESEARCH, PRACTICE OR POLICY**

- Together, these findings suggest that PA-related phenotypes may be present in a subset of patients with SSc and could contribute to the hypertension burden in this high-risk population, supporting consideration of PA screening in hypertensive patients with SSc, as is now recommended for all adult patients with hypertension.

## Introduction

 Systemic sclerosis (SSc) is a rare heterogeneous connective tissue disease,^1^ characterised by a high burden of hypertension (HTN) and cardiovascular disease (CVD).^2^,^8^ Despite the prevalence of HTN in SSc (31.2%–39.5%;^4 5^ ranging from 28% to 44% when stratified by disease subtype (limited vs diffuse, respectively)),^22^,^4 6^ the pathophysiological mechanisms underlying its development are not completely understood. Importantly, systemic HTN has been shown to be significantly associated with poor disease outcome in a Canadian SSc cohort.^2^

Primary aldosteronism (PA) is one of the most common secondary causes of HTN, estimated to affect 5%–14% of the general hypertensive population worldwide.^9^ While it confers greater cardiovascular risk than essential HTN, especially atrial fibrillation and stroke, it is highly modifiable, notably with mineralocorticoid receptor antagonists (MRA), or even curable with adrenal surgery.^9^ At present, however, PA is rarely tested for and substantially underdiagnosed in all hypertensive populations.^10^

There is limited evidence for a relationship between PA and autoimmune disorders including SSc. A study of 2319 patients with PA without previous autoimmune disease matched with 9276 patients with essential HTN found a significantly higher incidence of new onset autoimmune disease, including systemic lupus erythematosus (SLE), Sjögren’s disease, rheumatoid arthritis and idiopathic inflammatory myositis, in patients with PA versus essential HTN (HR 3.82, p<0.001);^11^ of note, only one patient with SSc was included in this study. Whether PA may contribute to HTN and CVD burden in SSc has never been explored. Here, we aimed to investigate the prevalence of HTN and PA in SSc, and to explore associations between markers of PA with SSc disease outcomes.

## Materials and methods

### Study participants

Steroid hormone data pertaining to these studied cohorts of SSc and HC have previously been published.^12^ The present study further explored these steroid hormone data via integration of blood pressure (BP) data and consideration of PA diagnosis. Consecutive adult patients fulfilling the 2013 American College of Rheumatology (ACR)/European Alliance of Associations for Rheumatology (EULAR) SSc classification criteria^13^ and followed in the Lille University Hospital Department of Internal Medicine and Clinical Immunology (Lille, France) were screened for study enrolment between June and October 2018. Healthy individuals were also recruited as healthy controls (HC) between February 2015 and January 2018, as part of the routine procedure of the Lille Hormonology laboratory to validate normal ranges for steroid hormone blood levels. Inclusion criteria for HC included: not using Renin–Angiotensin–Aldosterone System-interfering medications, not using glucocorticoids, normal potassium concentration and no personal history of HTN.

### Data collection and clinical assessment

Data were systematically prospectively collected in a standardised case report form for all SSc patients referred to our day-patient clinic. All participants underwent a comprehensive clinical evaluation performed within the same day. Data collected included patient’s demographics (including age, sex, weight, height) and SSc characteristics (including modified Rodnan skin score (mRSS) to assess skin fibrosis,^14^ biological results (including estimated glomerular filtration rate (eGFR), C-reactive protein levels and immunological profile (including antinuclear, anticentromere, antitopoisomerase I and anti-RNA polymerase III antibodies). Disease duration was defined as the time interval between the date of SSc diagnosis and the date of the study visit. The cutaneous subset of SSc was defined as limited (lc) or diffuse (dc) according to LeRoy’s criteria.^15^ Interstitial lung disease (ILD) was diagnosed on high-resolution chest CT scan. Pulmonary arterial hypertension (PAH) was diagnosed by right-heart catheterisation using the Seventh World Symposium haemodynamic definitions and classified into causative groups according to the updated clinical classification of pulmonary hypertension.^16^ A routine urine dipstick test was performed on patient’s visit; a urine protein-to-creatinine ratio (UPCR) was performed when positivity for at least 1+ protein was seen on urine dipstick. Scleroderma renal crisis (SRC) diagnosis was based on the presence of the ‘core set items to develop classification criteria for SRC’, as defined by Butler *et al*,^17^ which associate BP, kidney injury, thrombotic microangiopathy signs, target organ dysfunction and renal histopathology. Medication use was recorded including steroids, immunosuppressants, antihypertensives (Ca^2+^ channel blockers (CCB), angiotensin-converting enzyme inhibitors (ACEi), angiotensin II receptor blockers (ARB), beta blockers, diuretics, MRA), vasodilators (phosphodiesterase type 5 inhibitors, endothelin receptor antagonists, prostacyclin analogues and soluble guanylate cyclase stimulators), anticoagulants and antiplatelet agents.

HTN at baseline was defined as systolic BP (SBP) ≥140 mm Hg and/or diastolic BP (DBP) ≥90 mm Hg on the day of endocrine testing and on one prior clinical visit (median (IQR) follow-up: 1.1 (0.8, 1.5) years), or having a documented history of HTN in medical records. HTN at last follow-up visit was defined as SBP ≥140 mm Hg and/or DBP ≥90 mm Hg at both last visit and one prior clinical visit (median (IQR) follow-up: 1.1 (0.9, 1.2) years), or HTN assessed at baseline, or documented HTN in medical records. Drug-resistant HTN was defined as the use of three antihypertensives including one diuretic and BP ≥140/90 mm Hg, or the use of four antihypertensives, as per the 2017 American College of Cardiology/American Heart Association guidelines.^18^

SSc-related renal and cardiovascular outcomes were assessed at the last follow-up visit; renal outcomes included the presence of proteinuria (defined as UPCR>0.3 g/g), renal impairment (defined as eGFR<60 mL/min/1.73 m^2^) or SRC recorded at any time during the follow-up period; cardiovascular outcomes included stroke or myocardial infarction recorded at any time during the follow-up period.

HC were recruited by the Lille Hospital Hormonology laboratory. Prospectively collected data included: age, sex, height, weight, BP, medications, serum electrolyte panels, renal function and hormone levels.

### Biological sample collection, processing and storage

Venous blood was collected by venepuncture by a trained nurse, at the time of the study visit. Blood hormone levels were assessed in the morning (no more than 2 hours after getting up) in all study participants. As patients with SSc were tested during their routine clinical follow-up visit, some of them did not meet the optimal sampling conditions for assessing aldosterone and renin levels (ie, no interfering drugs, seated position, normal sodium diet and normokalaemia). This was documented in all patients. As per protocol, all the HC were sampled in optimal conditions.

### Blood markers of PA

Serum concentrations of potassium (mmol/L) were measured by indirect potentiometry. Assessment of hormone blood levels was performed by the Hormonology Laboratory of Lille University Hospital (Lille, France). Serum aldosterone (pmol/L) and 18-hydroxycortisol (18OHF; normal reference range (0.14–1.95 ng/mL; 0.39–5.40 nmol/L in follicular phase; 0.23–1.26 ng/mL; 0.64–3.49 nmol/L in luteal phase; 0.28–1.76 ng/mL; 0.78–4.88 nmol/L in males)) levels were measured by Liquid Chromatography-Mass Spectrometry (MS)/MS, Acquity UPLC Xevo TQ-XS, Waters). Plasma direct renin levels (mU/L) were assessed by chemiluminescence immunoassay (IDS-iSysVR IDS Direct Renin, IS-3400). The PA screening test, aldosterone:renin ratio (ARR), was considered positive if ARR>50 pmol/L:mU/L for individuals not taking interfering medications (including ACEi, ARB, diuretics, CCB (dihydropyridine) or MRA),^19^ or if ARR>30 pmol/L:mU/L for those on above-listed interfering medications or with another risk factor for a false negative ARR results (eg, serum K+levels < 3.5 mmol/L).^20^ Participants with a positive ARR were referred to endocrinology department for further evaluation.

### Statistical analysis

Data analysis and visualisation were performed using GraphPad Prism software (V.10.6.1). Descriptive statistics were used to report participant features, including proportion of individuals with HTN and drug-resistant HTN. Continuous data were summarised as mean (SD) or median (IQR) depending on data distribution. Categorical data were reported as frequencies (percentage). Differences in continuous variables between two groups were assessed using Mann-Whitney U test or t-test, depending on data distribution. Differences in categorical variables between two groups were assessed using χ^2^ or Fisher’s exact test, depending on the expected frequencies.

Regression modelling was used as our primary inferential tool in this study. Logistic regression models were used to investigate the clinical associations between markers of PA (exposure) and BP (outcome) in patients with SSc (baseline HTN~marker of PA). Univariable logistic regression models were used to investigate the associations between markers of PA (exposure) and SSc renal and cardiovascular outcomes (outcome) (SSc outcome~marker of PA). Multivariable linear and logistic regression modelling were fitted adjusting for the presence of potential confounding factors. A p value<0.05 was considered statistically significant.

### Patient and public involvement

None.

## Results

### Baseline characteristics

A total of 112 patients with SSc were included in this study (tables1  2). Briefly, at baseline median (IQR) age was 60 (49, 69) years, and 88 (79%) were female; 81 (72%) and 31 (28%) of the cohort had lcSSc and dcSSc subtypes, respectively. Median (IQR) mRSS was 4 (1, 9). Fifty-three (48%) and 17 (15%) had ILD and PAH, respectively (table 1). Median (IQR) period of follow-up was 5.1 (3.5, 6.1) years (table 2). The HC cohort comprised 48 individuals (median (IQR) age 42.5 (31, 57) years; 25 (52%) were female) (table 1).

**Table 1:** Characteristics of the SSc and HC cohorts at baseline.

	HC (n=48)	SSc (n=112)
Age (years), median (IQR)	42.5 (31, 57)	60 (49, 69)
Female sex, n (%)	25 (52%)	88 (79%)
Disease duration (years), median (IQR)	–	8 (3, 15)
Diffuse SSc, n (%)	–	31 (28%)
HTN	–	
SBP (mm Hg)*,* median (IQR)	119 (110, 128)	128 (118, 144)
DBP (mm Hg)*,* median (IQR)	70 (63, 77)	77 (69, 89)
HTN, n (%)	0 (0%)	41 (37%)
HTN* (BP assessed baseline and one prior visit)	0 (0%)	24 (21%)
History of HTN	0 (0%)	26 (23%)
Raynaud’s phenomenon,† n (%)	–	107 (99%)
Digital ulcers,‡ n (%)	–	59 (53%)
Calcinosis, n (%)	–	11 (11%)
mRSS, median (IQR)	–	4 (1, 9)
ILD,¶ n (%)	–	53 (48%)
PAH, n (%)	–	17 (15%)
SRC, n (%)	–	1 (0.8%)
ANA +, n (%)	–	107 (95%)
Antitopoisomerase I +, n (%)	–	24 (21%)
Anti-RNA polymerase III +, n (%)	–	3 (2.7%)
Anticentromere +, n (%)	–	63 (56%)
High CRP (>5 mg/L), n (%)	–	26 (23%)
High ESR (> 25 mm/hour),** n (%)	–	20 (18%)
Proteinuria††‡‡ n (%)	–	2 (1.7%)
eGFR<60 mL/min/1.73 m^2^, n (%)	–	13 (12%)
Hypokalemia§§,¶¶ n (%)		3 (2.6%)
Treatment, n (%)		
Corticosteroids use	–	46 (41%)
Immunosuppression use	–	32 (29%)
Vasodilator use***	–	18 (16%)
Anticoagulant	–	20 (18%)
Antiplatelet agent	–	33 (29%)
Ca2+ channel antagonist†††	–	60 (54%)
Angiotensin-converting enzyme inhibitor	–	14 (12%)
Angiotensin II receptor blockers	–	16 (14%)
Beta blocker	–	6 (5%)
Diuretic‡‡‡	–	22 (20%)
Mineralocorticoid receptor antagonists	–	4 (4%)
Hormone levels		
Aldosterone (pmol/L), median (IQR)	282.5 (146.1, 443.9)	281.3 (149.8, 502.6)
Renin (mIU/L), median (IQR)	21.05 (16.30, 39.10)	29.50 (15.08, 61.90)
ARR, median (IQR)	10.11 (7.69, 14.65)	10.71 (4.72, 18.54)
18OHF (ng/mL), median (IQR)	0.68 (0.52, 0.88)	0.81 (0.62, 1.16)

* Defined as SBP>140 mm Hg and/or DBP>90 mm Hg at both day of sampling and one prior clinical visit.

† Data missing for 4 patients.

‡ Past and/or present.

§ Data missing for 16 patients.

¶ Data missing for 1 patient.

** Data missing for 6 patients.

†† Defined as UPCR>0.3 g/g.

‡‡ Data missing for 10 patients.

§§ Defined as serum K+ levels < 3.5 mmol/L.

¶¶ Data missing for 2 patients.

*** PDE5 inhibitors, endothelin receptor antagonists, prostacyclin analogues or guanylate cyclase activators.

††† Dihydropyridine.

‡‡‡ Loop diuretics, thiazide diuretics and aldosterone antagonists.

ANA, antinuclear antibodies; ARR, aldosterone:renin ratio ; BP, blood pressure; CRP, C-reactive protein; DBP, diastolic blood pressure; eGFR, estimated glomerular filtration rate; ESR, erythrocyte sedimentation rate; HC, healthy controls; HTN, hypertension; ILD, interstitial lung disease; mRSS, modified Rodnan skin score; 18OHF, 18-hydroxycortisol; PAH, pulmonary arterial hypertension; PDE5, phosphodiesterase type 5; SBP, systolic blood pressure; SRC, scleroderma renal crisis; SSc, systemic sclerosis; UPCR, urine protein-to-creatinine ratio.

**Table 2:** Characteristics of the SSc cohort at the last follow-up visit.

	SSc (n=112)
Follow-up (years), median (IQR)	5.5 (3.5,6.1)
HTN	
SBP (mm Hg), median (IQR)	130 (120, 146)
DBP (mm Hg), median (IQR)	73 (64, 82)
HTN,* n (%)	55 (52%)
Renal outcomes,† n (%)	23 (22%)
Proteinuria‡§	8 (10%)
eGFR<60 mL/min/1.73 m^2^*	15 (14%)
SRC*	2 (1.9%)
CV outcomes¶, n (%)	6 (6%)
Stroke¶	4 (3.8%)
Myocardial infarction¶	2 (1.9%)

* Data missing for 6 patients.

† Data missing for 30 patients.

‡ Defined as UPCR > 0.3 g/g.

§ Data missing for 36 patients.

¶ Data missing for 7 patients.

** Defined as SBP>140 mm Hg and/or DBP>90 mm Hg at both day of sampling and one prior clinical visit.

CV, cardiovascular; DBP, diastolic blood pressure; eGFR, estimated glomerular filtration rate; HTN, hypertension; SBP, systolic blood pressure; SRC, scleroderma renal crisis; SSc, systemic sclerosis.

### Prevalence and management of HTN in SSc

At baseline, 37% (41/112) of patients with SSc had HTN, of whom 37% (15/41) had no documented diagnosis of HTN on medical records. A total of 3.6% (4/112) of the SSc cohort, corresponding to 9.7% (4/41) of the hypertensive SSc subset, had drug-resistant HTN. A total of 90 patients with SSc (80%), corresponding to 37 (90%) and 4 (100%) of those with HTN and drug-resistant HTN, respectively, were taking at least one interfering medication that can cause a false negative ARR results (online supplemental table 1). No significant difference in HTN prevalence was observed according to SSc disease subtype (limited SSc 37% vs diffuse SSc 35%, p=0.99) or the use of vasodilators (vasodilator 35% vs no vasodilator 37%, p=0.99). At the last follow-up visit, the prevalence of HTN increased to 52% (55/106) of the SSc cohort.

The HC cohort comprised only normotensive individuals, with median (IQR) SBP and DBP recorded at 119 (110, 128) mm Hg and 70 (63, 77) mm Hg, respectively.

### Profiles of blood biomarkers of PA in SSc

The prevalence of positive PA screening test results, based on ARR and accounting for interfering factors, was 8.0% (9/112) and 2% (1/51) in the SSc and HC cohorts. In the hypertensive SSc subset, the prevalence of positive PA screening test results was 12% (5/41). Among those with drug-resistant HTN (n=4), none had positive PA screening test results. Among the 9 SSc patients with a positive PA screening test results, none had 18OHF levels that were more than two times the upper limit of the normal reference range, with no significant differences in serum 18OHF levels observed between patients with and without a positive PA screening test results (median (IQR) 18OHF levels: in patients with ARR+: 0.89 (0.65–1.64) ng/mL; 2.48 (1.81;4.58) nmol/L ; in patients with normal ARR: 0.81 (0.61–1.15) ng/mL; 2.26 (1.70–3.20) nmol/L; p=0.51; online supplemental figure 1). Of note, none of the SSc patients with a positive PA screening test were taking beta blockers, known to cause false positive ARR results.^9^ Another subset of 9 patients with SSc harboured very low levels of renin (< 10 mU/L; mean (SD) aldosterone: 166 (139) pmol/L), while taking no interfering medications, and of whom 33% (3/9) had HTN.

We then explored the associations between biomarkers of PA and the presence of HTN cross-sectionally in the SSc cohort at baseline, using logistic regression modelling. No significant association was observed between levels of renin, aldosterone, ARR or 18OHF, with the presence of HTN in SSc in univariable analysis (online supplemental table 2**;** online supplemental figure 2), and these remained non-significant after adjusting for age, sex and ARR false negative risk (ie, use of ACEi, ARB, diuretics, CCB, or MRA and/or serum K+levels < 3.5 mmol/L) (online supplemental table 3). Similarly, while the prevalence of HTN was numerically higher in SSc patients with positive ARR results (55%, 5/9) compared with those without (35%, 36/103), no significant associations were observed between positive ARR results with the presence of HTN in SSc, in both univariable analysis and after adjusting for age and sex (online supplemental tables 2,3).

### Associations between biomarkers of PA and SSc-related outcomes

We next explored the relationships between markers of PA and SSc-related renal (whole cohort: n=23; in subset with positive ARR test results: n=3/9 (33%); in subset with no positive ARR test results: n=20/73 (27%)) and CV outcomes (whole cohort: n=6; in subset with positive ARR test results: n=0/9 (0%); in subset with no positive ARR test results: n=6/102 (5.8%)), as well as HTN (whole cohort: n=55; in subset with positive ARR test results: n=6/9 (66%); in subset with no positive ARR test results: n=49/97 (51%)) at last follow-up visit, using logistic regression modelling. Plasma renin levels were significantly associated with HTN at last follow-up visit, but not with SSc renal and cardiovascular outcomes, in univariable analysis (OR 1.007; 95% CI 1.001, 1.015; p=0.02; online supplemental table 7; online supplemental figure 3); however, this association attenuated after adjustment for sex, age and false negative risk (online supplemental table 8). No significant relationships were observed between aldosterone, ARR and 18OHF with SSc renal outcomes, cardiovascular outcomes and HTN at last follow-up visit in both univariable (online supplemental tables 4, 6, 7; online supplemental figure 3) and multivariable models (online supplemental tables 5, 8).

Similarly, no significant relationships were observed between positive ARR test results with SSc renal outcomes and HTN at last follow-up visit in both univariable (online supplemental tables 4, 6, 7) and multivariable models (online supplemental tables 5, 8). With regard to the relationships between positive ARR test results and CV outcomes, no significant difference in the prevalence of CV outcomes was observed between SSc patients with (0%, 0/9) and without (5.8%, 6/102) positive PA test results (p>0.99, Fisher’s exact test). It is noteworthy, however, that no logistic regression model could be fitted as no SSc patients who had positive PA test results had recorded any CV outcomes at the last follow-up, leading to the statistical case of complete separation.

## Discussion

Despite the HTN and CVD burden observed in SSc, knowledge surrounding their pathophysiology is incomplete. Whether PA may contribute to this burden remains to be elucidated. This study aimed to explore the prevalence of HTN in SSc and to determine the relationships between biomarkers of PA with HTN and SSc disease outcomes, particularly renal and cardiovascular. Systemic HTN was prevalent in our SSc cohort, estimated at 52% at the last follow-up visit. We observed a prevalence of positive PA screening test results of 12% in SSc patients with HTN. No significant associations were observed between biomarkers of PA with the presence of baseline HTN or with SSc renal and CV outcomes and HTN at follow-up.

In line with previous published studies,^2^,^46^ HTN was prevalent in our SSc cohort, estimated at 37% at baseline and increasing to 52% after a median period of follow-up of 5.1 years. No prior study reported on drug-resistant HTN in SSc. In our cohort, 9.7% of the hypertensive patients had drug-resistant HTN, a prevalence lower than in other connective tissue diseases such as SLE (16%).^21 22^ It is noteworthy that 37% of hypertensive SSc patients had no documented diagnosis of HTN on medical records at baseline.

Using ARR-based screening, we report for the first time a prevalence of positive PA screening results in SSc of 8% in the overall cohort, increasing to 12% among those with HTN. This prevalence is comparable to estimates in the general hypertensive population (5%–14%).^9^ Although mean ARR did not differ significantly between SSc and HC, a greater proportion of patients with SSc, particularly those with HTN, had positive ARR values, suggesting that PA-related phenotypes may be present in a subset of SSc patients. Four patients within the SSc cohort and one participant among HC had elevated ARR despite normal BP. Previous research has also demonstrated abnormal aldosterone profile in normotensive individuals, possibly reflecting early stages of biochemical aldosterone excess without the clinical phenotype.^23^ Further evaluation with aldosterone suppression tests will be needed to assess if there is underlying pathophysiology.

No significant associations were observed between biomarkers of PA and HTN, renal involvement or CV outcomes. However, interpretation is limited by the sample size, particularly the small number of patients with definitive CV endpoints. Larger prospective studies are needed to evaluate for potential associations between biomarkers of PA and CV and renal outcomes in SSc.

Additional study limitations warrant consideration. The presence of interfering medications, particularly ACE-I, ARB and diuretics, may cause false negative screening results by elevating renin. While a lower ARR threshold of 30 pmol/L:mU/L was used to account for ARR lowering by these interfering medications, it is still a very conservative threshold. ARR values as low as 15.2 pmol/mU (95% CI 4.9 to 20.0) have been reported in patients with PA on random antihypertensive medications.^24^ Of note, none of the four individuals with resistant hypertension in our cohort had a positive PA screening test. This speaks to the potential for multiple antihypertensives to obscure underlying hyperaldosteronism. A similar observation was made in the PATHWAY-2 trial for resistant hypertension, where secondary hypertension including PA was excluded at baseline, yet a subsequent substudy revealed that 25% of participants had inappropriately elevated aldosterone.^25^ A prospective study would therefore benefit from medication switching where safe to do so to unveil the true prevalence of PA.^26^ Another limitation relates to demographic differences between the SSc and HC groups, with the SSc participants being older and comprising a higher proportion of women. The ARR increases with age, meaning that lower ARR thresholds may be more sensitive for detecting aldosterone excess in younger individuals.^27^ In addition, the ARR tends to be higher in women than men, particularly in premenopausal women,^28^ although this sex difference is less likely to affect the interpretation of ARR in our SSc group given the median age of 60 years. Unfortunately, current guidelines do not provide advice for age- or sex-specific interpretation of the ARR. Finally, we were not able to report on the definite prevalence of confirmed PA as patients with an elevated ARR have not yet completed their full diagnostic workup.

In conclusion, this study confirmed the high prevalence of HTN in SSc and provides the first estimate of positive PA screening results in this population. Although biomarkers of PA were not associated with renal or CV outcomes in this relatively small cohort, the observed prevalence of abnormal ARR results suggests that PA screening should be performed in hypertensive patients with SSc, especially because of the deleterious cardiac and renal effects of aldosterone excess. This would align with recent clinical practice guidelines from the European Society of Cardiology^29^ and Endocrine Society,^19^ which suggest screening for PA in all adults with hypertension. The Endocrine Society guidelines also provide pragmatic guidance on interpreting and managing screening results when patients are unable to discontinue interfering medications. Whether treating aldosterone excess confers disease-specific benefits in patients with SSc remains uncertain. However, improvements in BP control, cardiovascular and renal outcomes and quality of life would be expected, as demonstrated in other patients with PA.^30^,^35^ Further research is needed to quantify the extent of benefit from identifying and treating PA in patients with SSc.

## Supplementary material

10.1136/rmdopen-2026-006930 online supplemental figure 1

10.1136/rmdopen-2026-006930 online supplemental figure 2

10.1136/rmdopen-2026-006930 online supplemental figure 3

10.1136/rmdopen-2026-006930 online supplemental file 1

10.1136/rmdopen-2026-006930 online supplemental file 2

## Data Availability

All data relevant to the study are included in the article or uploaded as supplementary information.
