# Supplementary figures and images for: Assessment of markers of primary aldosteronism in systemic sclerosis and their relationships with renal and cardiovascular outcomes
Source: RMD Open. 2026 Jul 15;12(3):e006930. doi: 10.1136/rmdopen-2026-006930 (PMC13374450; doi:10.1136/rmdopen-2026-006930)

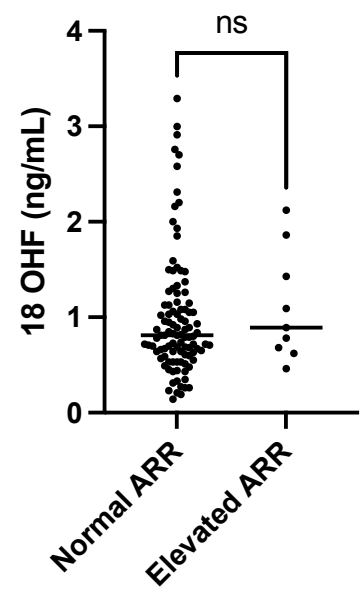

Supplement: online supplemental figure 1 [file rmdopen-12-3-s001.pdf]

**A**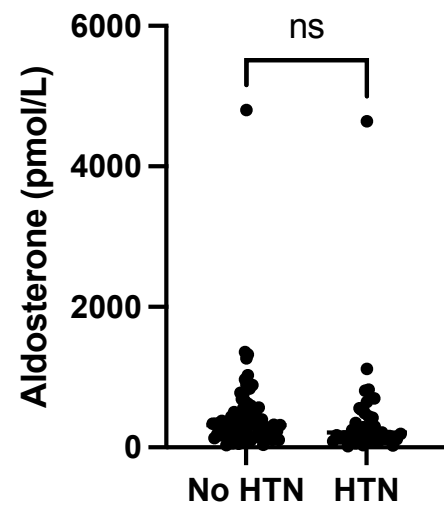**B**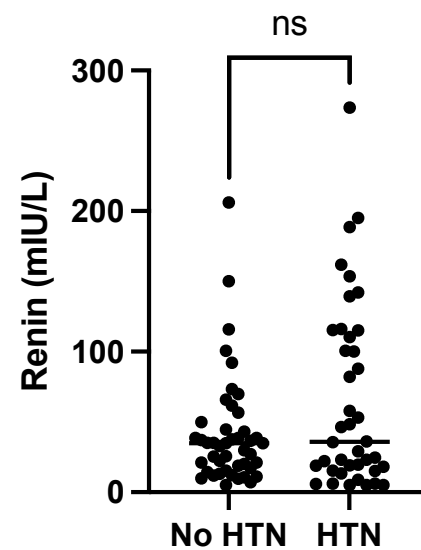**C**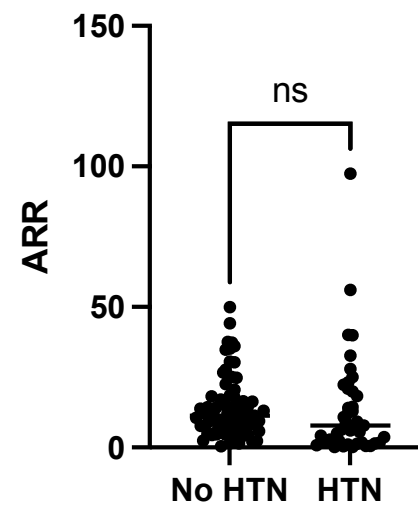**D**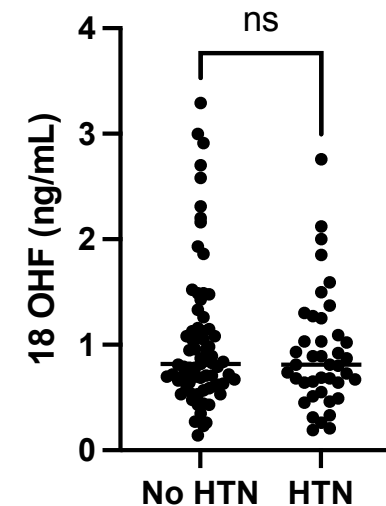

Supplement: online supplemental figure 2 [file rmdopen-12-3-s002.pdf]

**A**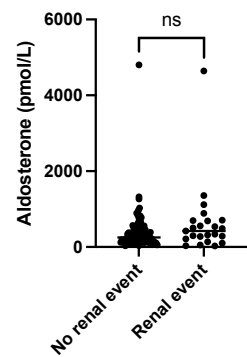**B**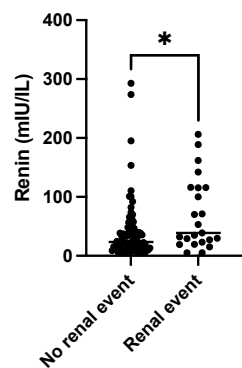**C**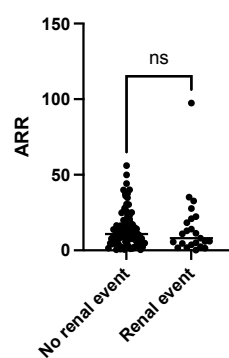**D**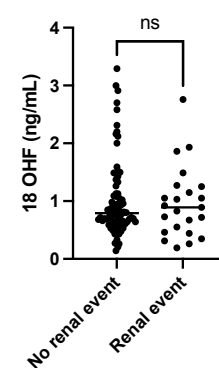**E**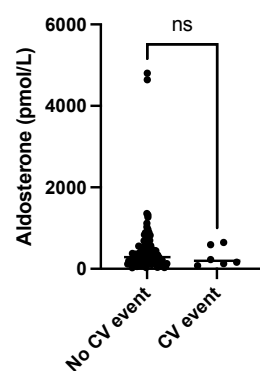**F**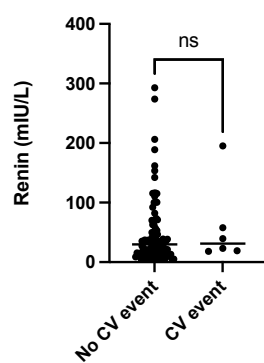**G**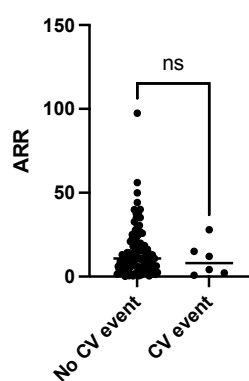**H**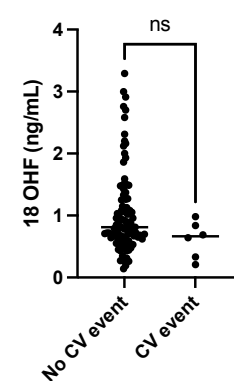**I**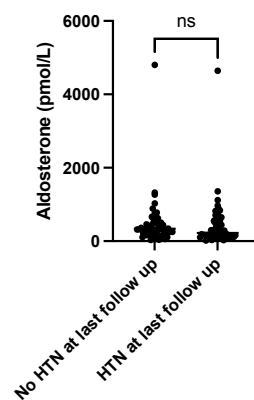**J**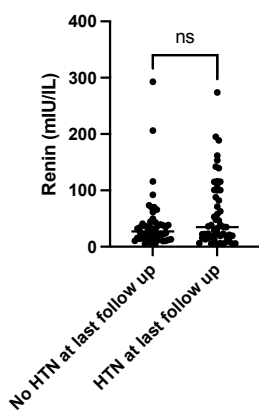**K**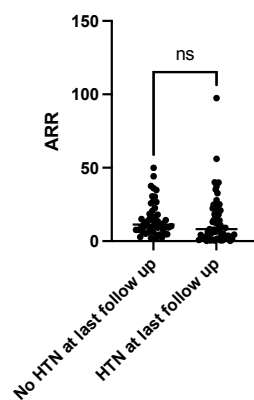**L**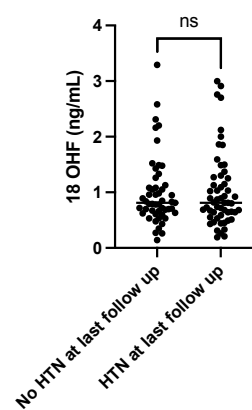

Supplement: online supplemental figure 3 [file rmdopen-12-3-s003.pdf]
