# Supplementary material for: Assessment of markers of primary aldosteronism in systemic sclerosis and their relationships with renal and cardiovascular outcomes
Source: RMD Open. 2026 Jul 15;12(3):e006930. doi: 10.1136/rmdopen-2026-006930 (PMC13374450; doi:10.1136/rmdopen-2026-006930)
Supplement: online supplemental file 1 [file rmdopen-12-3-s004.docx]

**SUPPLEMENTARY TABLES**

**Supplementary Table 1. Use of ARR interfering medication in the SSc cohort at baseline.**

|  | SSc  (N = 112) | SSc + HTN  (N = 41) | SSc + Resistant HTN  (N = 4) |
| --- | --- | --- | --- |
| Glucocorticoid*, n (%)* | 46 (41.1%) | 14 (34%) | 2 (50%) |
| Dose > 7.5 mg/d*, n (%)* | 22 (19.6%) | 7 (17.0%) | 1 (25%) |
| Diuretics*, n (%)* | 22 (19.6%) | 12 (29.3%) | 4 (100%) |
| ACEi*, n (%)* | 14 (12.5%) | 8 (19.5%) | 1 (25%) |
| ARB*, n (%)* | 16 (14.3%) | 13 (31.7%) | 3 (75%) |
| Ca2+ channel antagonist^#^, *n (%)* | 60 (53.6%) | 27 (65.8%) | 3 (75%) |
| MRA*, n (%)* | 4 (3.6%) | 0 (0%) | 0 (0%) |
| Progestin*, n (%)* | 5 (4.5%) | 2 (48.8%) | 0 (0%) |
| At least 1 interfering medication, *n (%)* | 90 (80.3%) | 37 (90.2%) | 4 (100%) |

**^#^** dihydropyridine

ACEi: angiotensin-converting enzyme inhibitor; ARB: angiotensin II receptor blockers; MRA: mineralocorticoid receptor antagonists ; SSc: systemic sclerosis

**Supplementary Table 2. Univariable associations between biomarkers of PA and presence of HTN in the SSc cohort at baseline.**

| ***Regression models*** | **Exposure variables** | **Estimate** | **SE** | **z value** | ***p*-value** | **OR** | **OR 95% IC** |
| --- | --- | --- | --- | --- | --- | --- | --- |
| **HTN** ~ **renin** | **Renin** | 0.0005 | 0.001 | 0.49 | 0.62 | 1.00 | 0.99 to 1.00 |
| **HTN** ~ **aldosterone** | **Aldosterone** | -0.0001 | 0.0003 | 0.53 | 0.72 | 0,99 | 0.99 to 1.00 |
| **HTN** ~ **ARR** | **ARR** | -0.003 | 0.01 | 0.21 | 0.83 | 0.99 | 0.97 to 1.02 |
| **HTN** ~ **18OHF** | **18OHF** | -0.31 | 0.33 | 0.94 | 0.34 | 0.73 | -0.99 to 0.31 |
| **HTN** ~ **abnormal ARR** | **Abnormal PA results** | 0.84 | 0.70 | 1.20 | 0.23 | 2.33 | 0.58 to 9.91 |

Odds ratios, 95% CI and *p*-values were derived from univariable logistic regression model.

ARR: aldosterone / renin ratio; CI: Confidence intervals; HTN: hypertension; OR: odds ratio; PA: primary aldosteronism; SSc: systemic sclerosis.

**Supplementary Table 3. Multivariable associations between biomarkers of PA and presence of HTN in the SSc cohort at baseline.**

| ***Regression models*** | **Exposure variables** | **Estimate** | **SE** | **z value** | ***p*-value** | **Adj. OR** | **Adj. OR 95% IC** |
| --- | --- | --- | --- | --- | --- | --- | --- |
| **HTN** ~ **renin** + sex + age + false neg risk | **Renin** | 0.0002 | 0.001 | 0.20 | 0.84 | 1.00 | (0.997, 1.003) |
|  | **Female sex** | 0.70 | 0.55 | 1.27 | 0.20 | 2.01 | (0.71, 6.24) |
|  | **Age** | 0.05 | 0.02 | 2.85 | **< 0.01** | 1.06 | (1.02, 1.10) |
|  | **False neg risk** | 1.04 | 0.48 | 2.15 | **0.03** | 2.84 | (1.13, 7.65) |
| **HTN** ~ **aldosterone** + sex + age + false neg risk | **Aldosterone** | -5.35 x 10^-5^ | 0.0003 | 0.15 | 0.88 | 1.00 | (0.999, 1.00) |
|  | **Female sex** | 0.68 | 0.54 | 1.27 | 0.19 | 1.99 | (0.71, 6.08) |
|  | **Age** | 0.05 | 0.02 | 2.79 | **< 0.01** | 1.06 | (1.02, 1.10) |
|  | **False neg risk** | 1.06 | 0.48 | 2.20 | **0.02** | 2.90 | (1.16, 7.82) |
| **HTN** ~ **ARR** + sex + age + false neg risk | **ARR** | -0.001 | 0.01 | 0.10 | 0.92 | 1.00 | (0.97, 1.03) |
|  | **Female sex** | 0.69 | 0.54 | 1.26 | 0.20 | 1.99 | (0.71, 6.10) |
|  | **Age** | 0.05 | 0.02 | 2.85 | **< 0.01** | 1.06 | (1.02, 1.10) |
|  | **False neg risk** | 1.06 | 0.48 | 2.20 | **0.02** | 2.87 | (1.16, 7.69) |
| **HTN** ~ **18OHF** + sex + age + false neg risk | **18OHF** | -0.47 | 0.36 | 1.30 | 0.19 | 0.62 | (0.30, 1.27) |
|  | **Female sex** | 0.71 | 0.55 | 1.30 | 0.19 | 2.03 | (0.70, 5.93) |
|  | **Age** | 0.06 | 0.02 | 2.97 | **< 0.01** | 1.06 | (1.02, 1.10) |
|  | **False neg risk** | 1.02 | 0.48 | 2.13 | **0.03** | 2.785 | (1.08, 7.45) |
| **HTN** ~ **abnormal ARR** + sex + age | **Abnormal PA results** | 0.95 | 0.76 | 1.25 | 0.21 | 2.59 | (0.59, 12.66) |
|  | **Female sex** | 0.43 | 0.53 | 0.81 | 0.42 | 1.53 | (0.56, 4.52) |
|  | **Age** | 0.06 | 0.02 | 2.98 | **< 0.01** | 1.06 | (1.02, 1.099) |

Adjusted odds ratios, 95% CI and *p*-values were derived from multivariable logistic regression model adjusting for age, sex and ARR false negative risk (i.e., use of ACEi, ARB, diuretics, CCB, or MRA and / or serum K+ levels < 3.5 mmol/l).

Adj.: adjusted; ARR: aldosterone / renin ratio; CI: Confidence intervals; HTN: hypertension; OR: odds ratio; PA: primary aldosteronism; SSc: systemic sclerosis.

**Supplementary Table 4. Univariable associations between biomarkers of PA and renal outcomes in the SSc cohort at last follow-up.**

| ***Regression models*** | **Exposure variables** | **Estimate** | **SE** | **z value** | ***p*-value** | **OR** | **OR 95% CI** |
| --- | --- | --- | --- | --- | --- | --- | --- |
| **Renal outcomes** ~ **renin** | **Renin** | 4.13 x 10^-5^ | 0.001 | 0.03 | 0.97 | 1.00 | (0.99,1.00) |
| **Renal outcomes** ~ **aldosterone** | **Aldosterone** | 0.0004 | 0.0003 | 1.36 | 0.17 | 1.00 | (0.99,1.00) |
| **Renal outcomes** ~ **ARR** | **ARR** | 0.003 | 0.02 | 0.18 | 0.86 | 1.00 | (0.96,1.03) |
| **Renal outcomes** ~ **18OHF** | **18OHF** | -0.11 | 0.38 | 0.29 | 0.77 | 0.89 | (0.40,1.80) |
| **Renal outcomes** ~ **abnormal ARR** | **Abnormal PA results** | 0.64 | 0.75 | 0.85 | 0.41 | 1.90 | (0.38,7.89) |

Odds ratios, 95% CI and *p*-values were derived from univariable logistic regression model.

ARR: aldosterone / renin ratio; CI: Confidence intervals; HTN: hypertension; OR: odds ratio; PA: primary aldosteronism; SSc: systemic sclerosis.

**Supplementary Table 5. Multivariable associations between biomarkers of PA and renal outcomes in the SSc cohort at last follow-up.**

| ***Regression models*** | **Exposure variables** | **Estimate** | **SE** | **z value** | ***p*-value** | **Adj. OR** | **Adj. OR 95% CI** |
| --- | --- | --- | --- | --- | --- | --- | --- |
| **Renal outcomes** ~ **renin** + sex + age + false neg risk | **Renin** | 0.0002 | 0.001 | 0.14 | 0.89 | 1.00 | (0.996, 1.003) |
|  | **Female sex** | 1.42 | 0.80 | 1.77 | 0.08 | 4.13 | (0.86, 19.90) |
|  | **Age** | 0.01 | 0.02 | 0.50 | 0.61 | 1.01 | (0.97, 1.05) |
|  | **False neg risk** | 0.60 | 0.55 | 1.10 | 0.27 | 1.82 | (0.62, 5.32) |
| **Renal outcomes** ~ **aldosterone** + sex + age + false neg risk | **Aldosterone** | 0.0004 | 0.0003 | 1.21 | 0.22 | 1.00 | (0.997, 1.001) |
|  | **Female sex** | 1.35 | 0.80 | 1.69 | 0.09 | 3.84 | (0.81, 18.27) |
|  | **Age** | 0.02 | 0.02 | 0.83 | 0.40 | 1.02 | (0.97, 1.06) |
|  | **False neg risk** | 0.49 | 0.55 | 0.90 | 0.36 | 1.64 | (0.56, 4.82) |
| **Renal outcomes** ~ **ARR** + sex + age + false neg risk | **ARR** | -0.0002 | 0.02 | 0.02 | 0.99 | 1.00 | (0.97, 1.03) |
|  | **Female sex** | 1.40 | 0.79 | 1.77 | 0.08 | 4.06 | (0.86, 19.25) |
|  | **Age** | 0.01 | 0.02 | 0.51 | 0.61 | 1.01 | (0.97, 1.05) |
|  | **False neg risk** | 0.61 | 0.54 | 1.13 | 0.26 | 1.84 | (0.64, 5.31) |
| **Renal outcomes** ~ **18OHF** + sex + age + false neg risk | **18OHF** | -0.15 | 0.40 | 0.38 | 0.70 | 0.86 | (0.39, 1.88) |
|  | **Female sex** | 1.41 | 0.79 | 1.79 | 0.07 | 4.11 | (0.87, 1.41) |
|  | **Age** | 0.01 | 0.02 | 0.54 | 0.59 | 1.01 | (0.97, 1.05) |
|  | **False neg risk** | 0.60 | 0.54 | 1.11 | 0.27 | 1.82 | (0.63, 5.25) |
| **Renal outcomes** ~ **abnormal ARR** + sex + age | **Abnormal PA results** | 0.57 | 0.76 | 0.74 | 0.46 | 1.76 | (0.4, 7.85) |
|  | **Female sex** | 1.27 | 0.78 | 1.61 | 0.10 | 3.57 | (0.77, 16.65) |
|  | **Age** | 0.01 | 0.02 | 0.64 | 0.52 | 1.01 | (0.87, 1.05) |

Adjusted odds ratios, 95% CI and *p*-values were derived from multivariable logistic regression model adjusting for age, sex and ARR false negative risk (i.e., use of ACEi, ARB, diuretics, CCB, or MRA and / or serum K+ levels < 3.5 mmol/l).

Adj.: adjusted; ARR: aldosterone / renin ratio; CI: Confidence intervals; HTN: hypertension; OR: odds ratio; PA: primary aldosteronism; SSc: systemic sclerosis.

**Supplementary Table 6. Univariable associations between biomarkers of PA and CV outcomes in the SSc cohort at last follow-up.**

| **Exposure variables** | **Estimate** | **SE** | **z value** | ***p*-value** | **OR** | **OR 95% CI** |
| --- | --- | --- | --- | --- | --- | --- |
| **Renin** | -0.0005 | 0.003 | 0.14 | 0.86 | 0.9995 | (0.98, 1.00) |
| **Aldosterone** | -0.001 | 0.002 | 0.64 | 0.45 | 0.999 | (0.99, 1.00) |
| **ARR** | 0.04 | 0.04 | 0.74 | 0.38 | 0.97 | (0.87, 1.03) |
| **18OHF** | -2.17 | 1.42 | 1.53 | 0.05 | 0.11 | (0.005, 1.03) |
| **Abnormal PA results** | **^#^** | **^#^** | **^#^** | **^#^** | **^#^** | **^#^** |

Odds ratios, 95% CI and *p*-values were derived from univariable logistic regression model.

^#^ No univariable logistic regression model could be fitted as no SSc patients who had abnormal PA screening test results had recorded any CV outcomes at the last follow-up, leading to the statistical case of complete separation.

ARR: aldosterone / renin ratio; CI: Confidence intervals; OR: odds ratio; PA: primary aldosteronism; SSc: systemic sclerosis.

**Supplementary Table 7. Univariable associations between biomarkers of PA and HTN in the SSc cohort at last follow-up.**

| ***Regression models*** | **Exposure variables** | **Estimate** | **SE** | **z value** | ***p*-value** | **OR** | **OR 95% CI** |
| --- | --- | --- | --- | --- | --- | --- | --- |
| **HTN** ~ **renin** | **Renin** | 0.007 | 0.004 | 1.74 | **0.02** | 1.007 | (1.001,1.015) |
| **HTN** ~ **aldosterone** | **Aldosterone** | -0.0001 | 0.0003 | 0.39 | 0.69 | 0.99 | (0.99,1.00) |
| **HTN** ~ **ARR** | **ARR** | -0.004 | 0.01 | 0.28 | 0.78 | 0.99 | (0.97,1.02) |
| **HTN** ~ **18OHF** | **18OHF** | 0.05 | 0.30 | 0.18 | 0.86 | 1.06 | (0.58,1.93) |
| **HTN** ~ **abnormal ARR** | **Abnormal PA results** | 0.67 | 0.73 | 0.91 | 0.35 | 1.96 | (0.49,9.70) |

Odds ratios, 95% CI and *p*-values were derived from univariable logistic regression model.

ARR: aldosterone / renin ratio; CI: Confidence intervals; HTN: hypertension; OR: odds ratio; PA: primary aldosteronism; SSc: systemic sclerosis.

**Supplementary Table 8. Multivariable associations between biomarkers of PA and HTN in the SSc cohort at last follow-up.**

| ***Regression models*** | **Exposure variables** | **Estimate** | **SE** | **z value** | ***p*-value** | **Adj. OR** | **Adj. OR 95% CI** |
| --- | --- | --- | --- | --- | --- | --- | --- |
| **HTN** ~ **renin** + sex + age + false neg risk | **Renin** | -0.005 | 0.004 | 1.38 | 0.17 | 0.9997 | (0.986, 1.002) |
|  | **Female sex** | -0.26 | 0.49 | 0.52 | 0.60 | 0.77 | (0.29, 2.04) |
|  | **Age** | -0.03 | 0.02 | 1.79 | 0.07 | 0.97 | (0.93, 1.003) |
|  | **False neg risk** | -0.37 | 0.45 | 0.79 | 0.43 | 0.70 | (0.29, 1.70) |
| **HTN** ~ **aldosterone** + sex + age + false neg risk | **Aldosterone** | 7.17 x 10^-5^ | 0.0003 | 0.23 | 0.82 | 1.00 | (0.999, 1.00) |
|  | **Female sex** | -0.22 | 0.49 | 0.45 | 0.66 | 0.80 | (0.31, 2.09) |
|  | **Age** | -0.03 | 0.02 | 1.79 | 0.07 | 0.97 | (0.94, 1.00) |
|  | **False neg risk** | -0.60 | 0.44 | 1.38 | 0.17 | 0.55 | (0.23, 1.28) |
| **HTN** ~ **ARR** + sex + age + false neg risk | **ARR** | 0.002 | 0.01 | 0.12 | 0.90 | 1.00 | (0.97, 1.03) |
|  | **Female sex** | -0.21 | 0.49 | 0.43 | 0.66 | 0.80 | (0.31, 2.11) |
|  | **Age** | -0.03 | 0.02 | 1.87 | 0.06 | 0.97 | (0.94, 1.002) |
|  | **False neg risk** | -0.59 | 0.43 | 1.36 | 0.17 | 0.56 | (0.24, 1.29) |
| **HTN** ~ **18OHF** + sex + age + false neg risk | **18OHF** | -0.01 | 0.31 | 0.04 | 0.96 | 0.99 | (0.53, 1.82) |
|  | **Female sex** | -0.20 | 0.48 | 0.41 | 0.67 | 0.81 | (0.21, 2.11) |
|  | **Age** | -0.03 | 0.02 | 1.86 | 0.06 | 0.97 | (0.94, 1.00) |
|  | **False neg risk** | -0.89 | 0.43 | 1.37 | 0.17 | 0.55 | (0.23, 1.29) |
| **HTN** ~ **abnormal ARR** + sex + age | **Abnormal PA results** | -0.76 | 0.77 | 0.99 | 0.32 | 0.47 | (0.10, 2.10) |
|  | **Female sex** | -0.06 | 0.48 | 0.12 | 0.90 | 0.94 | (0.67, 2.40) |
|  | **Age** | -0.03 | 0.02 | 2.06 | **0.04** | 0.97 | (0.94, 0.99) |

Adjusted odds ratios, 95% CI and *p*-values were derived from multivariable logistic regression model adjusting for age, sex and ARR false negative risk (i.e., use of ACEi, ARB, diuretics, CCB, or MRA and / or serum K+ levels < 3.5 mmol/l).

Adj.: adjusted; ARR: aldosterone / renin ratio; CI: Confidence intervals; HTN: hypertension; OR: odds ratio; PA: primary aldosteronism; SSc: systemic sclerosis.
