# Supplementary material for: Assessment of markers of primary aldosteronism in systemic sclerosis and their relationships with renal and cardiovascular outcomes
Source: RMD Open. 2026 Jul 15;12(3):e006930. doi: 10.1136/rmdopen-2026-006930 (PMC13374450; doi:10.1136/rmdopen-2026-006930)
Supplement: online supplemental file 2 [file rmdopen-12-3-s005.docx]

**SUPPLEMENTARY FIGURE LEGENDS**

**Supplementary Figure 1. Serum 18OHF levels stratified by ARR test results in SSc cohort at baseline.**

Serum levels of 18OHF in patients with SSc stratified by the presence of abnormal PA screening test results.

*H*orizontal bar represents the median. *p*-values were derived from Mann-Whitney U tests.

18OHF: 18-Hydroxycortisol; ARR: aldosterone / renin ratio; ns: not significant.

**Supplementary Figure 2. Associations between blood markers of PA and HTN in SSc cohort at baseline.**

Blood levels of **A)** aldosterone, **B)** renin, **C)** ARR, and **D)** 18OHF, in patients with SSc stratified by the presence of HTN.

*Panels A-D*: horizontal bar represents the median. *p*-values were derived from Mann-Whitney U tests.

18OHF: 18-Hydroxycortisol; ARR: aldosterone / renin ratio; HTN: hypertension; ns: not significant.

**Supplementary Figure 3. Associations between blood markers of PA with SSc outcomes and HTN at last follow-up visit.**

Blood levels of: **A)** aldosterone, **B)** renin, **C)** ARR, and **D)** 18OHF in patients with SSc stratified by the presence of renal outcomes at the last follow-up visit; **E)** aldosterone, **F)** renin, **G)** ARR, and **H)** 18OHF in patients with SSc stratified by the presence of cardiovascular outcomes at the last follow-up visit; **I)** aldosterone, **J)** renin, **K)** ARR, and **L)** 18OHF in patients with SSc stratified by the presence of HTN at the last follow-up visit.

*Panels A-L*: horizontal bar represents the median. *p*-values were derived from Mann-Whitney U tests.

18OHF: 18-Hydroxycortisol; ARR: aldosterone / renin ratio; CV: cardiovascular; HTN: hypertension; ns: not significant.

^*^ *p*-value < 0.05
